# Temperature-dependent birefringence of lithium triborate, LBO in the THz regime

**DOI:** 10.1038/s41598-017-08626-2

**Published:** 2017-08-14

**Authors:** Kechao Song, Zhen Tian, Weili Zhang, Mingwei Wang

**Affiliations:** 1 Institute of Modern Optics, Nankai University, Key Laboratory of Optical Information Science and Technology, Ministry of Education, Tianjin, 300350 China; 2 Center for Terahertz Waves and School of Precision Instrument and Optoelectronics Engineering, Tianjin University, and Key Laboratory of Optoelectronics Information and Technology, Ministry of Education of China, Tianjin, 300072 China; 3 School of Electrical and Computer Engineering, Oklahoma State University, Stillwater, OK 74078 USA

## Abstract

Optical properties of lithium triborate (LBO) in the terahertz regime (0.2–2 THz) were characterized using broadband terahertz time-domain spectroscopy. The frequency dependence of refractive index and absorption coefficient of the LBO crystal was experimentally investigated over the temperature range of 77–297 K, which the experimental results indicated that LBO has very low optical absorption coefficient at terahertz frequencies especially for the beam polarization along the crystal’s principal dielectric axis X. Moreover, a giant birefringence was observed, and the refractive index difference between the axis X and Z gradually decreased with decreasing temperature, which is attributed to the behavior of the TO phonon modes of B_1_ and B_2_ symmetries at low frequencies at different temperatures. As potential applications, LBO can be exemplarily used as terahertz wave shapers, beam splitters, terahertz wave plates, circular polarizers and other polarization devices.

## Introduction

In recent years, terahertz wave has received extensive attention and reveals potentials in numerous applications, particularly in homeland security and fundamental research^1–3^. Besides terahertz sources and detectors, the development of terahertz components for manipulating polarization, for example wave plates, has been lagging at these frequencies. It is expected that terahertz polarization devices will profit from birefringent materials and components. Several materials with terahertz birefringence have been reported^4–7^, paving the way to developing birefringence based functional terahertz devices including wave plates^8, 9^, filters^10^ and phase shifters^11, 12^.

Lithium triborate (LiB_3_O_5_, LBO) is known to be an orthorhombic negative biaxial crystal belonging to the *mm2* point symmetry group (${C}_{2\upsilon }$), coinciding with space group Pna2_1_ (${C}_{2\upsilon }^{9}$)^13^. The crystal principal dielectric axes X, Y, Z (${n}_{X} < {n}_{Y} < {n}_{Z}$) were found to be parallel to the crystallographic axes a, c, b^14^. The lithium triborate (LBO) crystal^15^ is widely used for optical frequency doubling, optical parametric oscillator (OPO), optical parametric amplification (OPA)^16^, and other nonlinear optical processes due to its extremely low linear optical absorption, large birefringence, moderate nonlinear coefficients, and high damage threshold (45 GW · cm^−2^ for 1 ns pulses at 1.064 μm)^17^. In addition, it is nonhygroscopic and chemically stable with good mechanical properties and has a high melting point of 1107 K^18^. These outstanding merits make LBO a potential terahertz source^19^. The properties of LBO in the infrared and far infrared regions above 100 cm^−1^ (3 THz) have been studied by Raman and infrared reflection spectroscopy^20–23^. However, the optical properties of LBO crystal in the terahertz range have not been fully characterized and systematically studied, especially for different temperatures^24–27^.

In this letter, we investigated the optical properties of LBO in the terahertz regime (0.2–2 THz) using a broadband terahertz time-domain spectrometer (THz-TDS). The crystal orientation and frequency dependence of refractive index and absorption coefficient were experimentally investigated over the temperature range of 77–297 K for the first time to our knowledge. Our experimental results showed that LBO has a giant birefringence and low optical absorption coefficient especially for the wave polarizing along the X-axis which makes it suitable for terahertz wave plates, wave shapers, beam splitters, circular polarizers and other polarization devices. Furthermore, we studied the optical properties dependence on the crystal orientation and temperature. Finally, we analyzed the experimental results from the perspective of crystal phonon modes with Kurosawa relation and the classical pseudo-harmonic phonon model and demonstrated the validity of our results.

## Results

**Figure 1:**
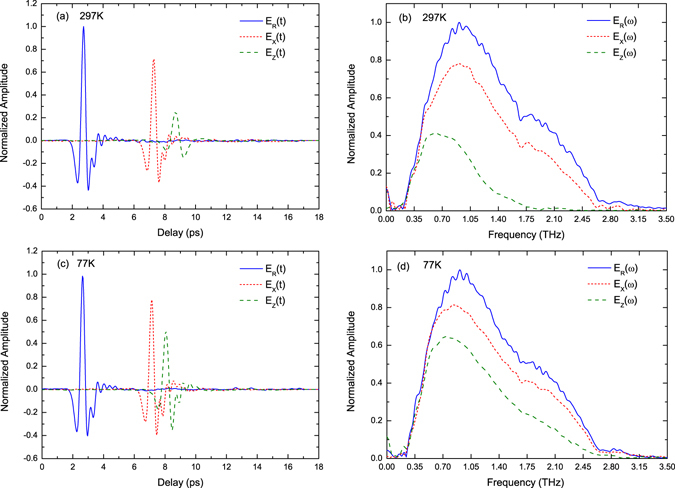
Measured time and frequency domain waveforms of the reference pulse and sample pulse at 297 K and 77 K. The blue solid line, the red short dashed line and the olive dashed line represent reference pulse, sample pulses polarizing along X-axis and Z-axis, respectively. (**a**,**c**) Temporal waveforms at 297 K and 77 K, respectively. (**b**,**d**) Spectra in the range of 0 to 3.5 THz at 297 K and 77 K, respectively.

According to the well-known refractive index relationship ${{\rm{n}}}_{{\rm{Z}}} > {{\rm{n}}}_{{\rm{Y}}} > {{\rm{n}}}_{{\rm{X}}}$ in the main transparency window, the refractive index difference between the crystal principal dielectric axes Z and X is maximal which is more attractive for terahertz device applications. Therefore, the absorption coefficients and refractive indices along the two directions in the terahertz region are investigated. In the experiment, the propagation of the incident terahertz pulse is along the Y-axis, and the polarization is along the Z-axis and X-axis, respectively. The transmitted temporal waveforms and the corresponding spectra of the 1.07 mm thick LBO crystal and reference signals in the case of the incident pulse with its polarization along the Z-axis and X-axis were observed at room temperature 297 K and the liquid nitrogen temperature 77 K, respectively, as shown in Fig. 1. Figure 1(a) and (c) are the transmitted terahertz waveforms in the time domain at 297 K and 77 K, respectively. As can be seen in Fig. 1(a), the relative time delay of between the reference and sample signal polarizing along the Z-axis is nearly 6 ps at 297 K. Through the relative time delay and the thickness of the LBO sample, we estimate the average refractive index of the LBO crystal in the Z-axis to be approximately 2.68. Comparing to Fig. 1(c), the relative time delay between the reference and sample signal polarizing along the Z-axis decreases to 5.5 ps, which indicates the refractive index in the corresponding direction declines by 0.14 on average at low temperature 77 K, while the refractive index of the X-axis almost keeps unchanged. From the spectral information in Fig. 1(b) and (d), we can see that the main frequency range of the transmitted terahertz pulse exists in 0.2–2 THz and the absorption at low temperature 77 K is lower than that at room temperature 297 K. But for the input pulse with its polarization along the Z-axis at 297 K, the effective frequency range is less than 1.75 THz, which could be caused by the relative strong absorption in the direction.

**Figure 2:**
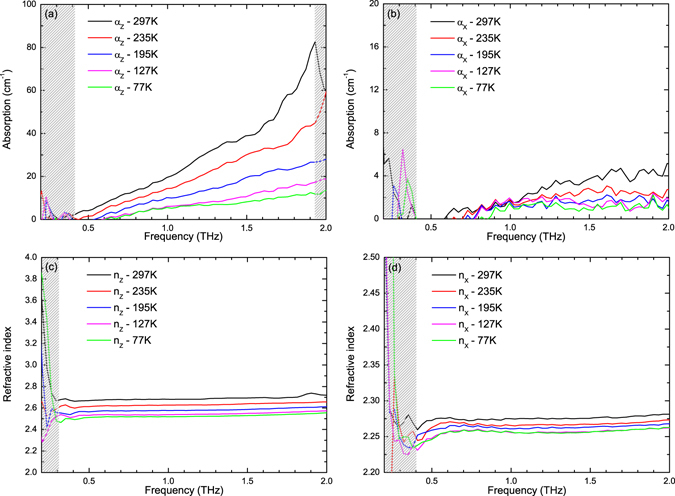
(**a**,**b**) Measured absorption coefficients *α*_*Z*_ and *α*_*X*_ from 0.2 to 2.0 THz. (**c**,**d**) Measured refractive indices *n*_*Z*_ and *n*_*x*_ from 0.2 to 2.0 THz.

To further study the temperature-dependent terahertz properties of LBO crystals, we carried out the experiments at 297 K, 235 K, 195 K, 127 K and 77 K, respectively. The corresponding transmitted temporal waveforms and spectra at each temperature can be found as Supplementary Fig. S2. The absorption coefficients and refractive indices were measured for the incident terahertz pulse polarization along the X-axis and Z-axis direction, as shown in Fig. 2. Figure 2(a) and (b) display the curves of α_Z_ and α_X_ changing with frequency at different temperatures, respectively. The gray slash regions in Fig. 2 and the following figures are not accurate region due to the limitation of the THz-TDS dynamic range. The absorption coefficient curves α_X_ and α_Z_ at all temperatures increase monotonically with frequency and do not appear any absorption peaks due to the fact that the lowest transverse optical phonon vibration mode is located at a higher frequency 3.3 THz at 300 K^20^. The absorption coefficients α_Z_ and α_X_ decrease with dropping temperature. This may be related to the following behaviors: (1) the low-frequency optical phonon modes B_1T_ and B_2T_ increase monotonically with decreasing temperature^20^; (2) the Raman bands become narrow and blue shift at low-frequencies^21^. The absorption coefficient in the X-axis is not over 5 cm^−1^ in the range of 0.2–2 THz which is far less than that in the Z-axis. Compared with other nonlinear optical crystals, the absorption coefficient α_X_ is the lowest absorption in the 0.2–2 THz range among the known nonlinear crystals^24^. Figure 2(c) and (d) show the refractive indices change in the two directions with frequency at different temperatures. n_X_ and n_Z_ increase slowly with frequency in the range of 0.3–2 THz without large dispersion or anomalous dispersion ascribed to the fact that these frequencies are sufficiently far from the phonon peak associated with the resonance. The maximum refractive index difference ${\rm{\Delta }}n={n}_{Z}-{n}_{X}\approx 0.42$ can be obtained at 297 K and drops with decreasing temperature. For the Z-axis polarization, when temperature decreases from 297 K to 77 K, the refractive index decreases by 0.21, while this value only decreases by 0.02 for the X-axis polarization. The measured refractive indices and absorption coefficients of the LBO crystal at room temperature in our experiment are almost in line with the previous experiments in refs 24 and 26, except for a controversy on polarization and propagation of the incident terahertz pulse. In these previous work, the absorption coefficients and refractive indices in the X-axis and Z-axis were obtained on the condition of the incident pulse propagation along the corresponding direction, while we consider these to be obtained on the condition of the incident pulse polarization along the corresponding direction. According to our experimental measurements with respect to temperature and crystal orientation, we can see that both of the absorption coefficient and refractive index decrease with lowering temperature in the two directions. However, the absorption coefficient and refractive index of the LBO crystal in the Z-axis appear to be more sensitive to temperature than those in the X-axis.

## Discussion

**Figure 3:**
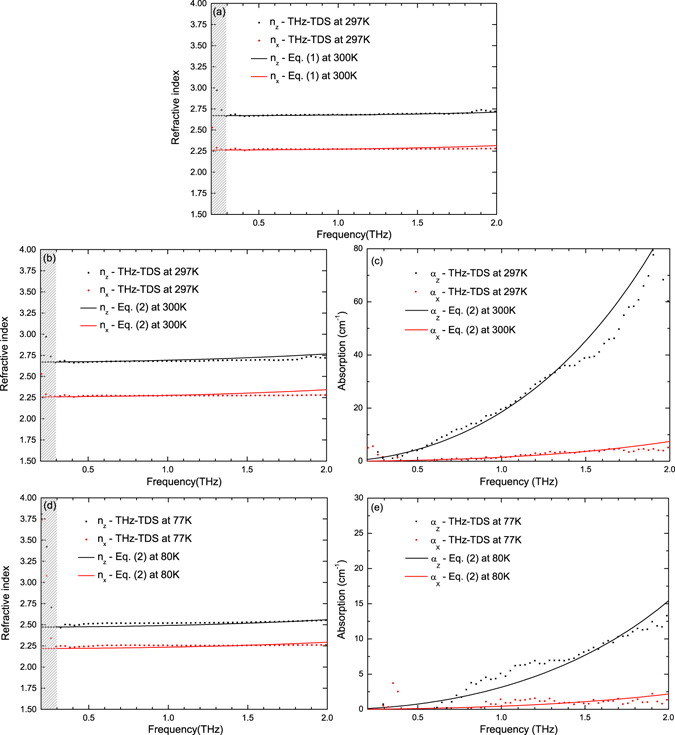
(**a**) Experimental results *n*_*Z*_ (black solid square) and *n*_*X*_ (red solid circle) from 0.2 to 2.0 THz at 297 K; Fitting results of *n*_*Z*_ (black line) and *n*_*X*_ (red line) from 0.2 to 2.0 THz at 300 K based on Eq. (1). (**b**,**d**) Experimental results *n*_*Z*_ (black solid square) and *n*_*X*_ (red solid circle) from 0.2 to 2.0 THz at 297 K and 77 K, respectively; Fitting results *n*_*Z*_ (black line) and *n*_*X*_ (red line) from 0.2 to 2.0 THz based on Eq. (2) at 300 K and 80 K, respectively. (**c**,**e**) Experimental results *α*_*Z*_ (black solid square) and *α*_*X*_ (red solid circle) from 0.2 to 2.0 THz at 297 K and 77 K; Fitting results *α*_*Z*_ (black line) and *α*_*X*_ (red line) from 0.2 to 2.0 THz based on Eq. (2) at 300 K and 80 K, respectively.

To further explain why refractive index and absorption coefficient in the X- and Z-axis change with temperatures, we discuss the optical properties of the LBO crystal from the aspect of optical phonon. Since LBO is a non-centrosymmetric polar crystal^28^, we need to figure out the polar lattice vibration modes with infrared activity according to the dynamics theory of lattice vibration^29, 30^. Based on the group theory, the space group of the LBO crystal is Pna2_1_ (${C}_{2\upsilon }^{9}$). At zero wave vector, the optical phonon modes have the following form^20, 22^: $26{A}_{1}+27{A}_{2}+26{B}_{1}+26{B}_{2}$, where all of the optical modes are Raman active, and A_1_, B_1_ and B_2_ are also infrared active. The polarizations of A_1_, B_1_ and B_2_ are parallel to the crystal axis c, a, b, respectively, which correspond to the crystal principal dielectric axes Y, X, Z. Based on this, we study the behavior of optical vibration modes B_1_, B_2_ at different temperatures, which is relevant to terahertz optical properties in the direction of the X- and Z-axis, respectively. As we could not find enough Raman or IR spectra data of LBO at 297–77 K, we discuss the changes from the qualitative perspective. The Raman spectra data of LBO at room temperature 300 K and liquid nitrogen temperature 80 K are cited from refs 20 and 22, respectively. The influence of 3 K temperature difference between the data and our experiments can be ignored since it can hardly affect the optical properties of LBO. Table 1 is the low frequency phonon modes B_1_, B_2_ of Raman spectrum of the LBO crystal at 300 K and 80 K, respectively, and the complete table can be seen as Supplementary Table S1. The refractive index of LBO at corresponding temperature can be calculated according to the data in Table 1 combining with the Kurosawa equation^31^:

1 $${\varepsilon }_{\alpha }(\omega )={\varepsilon }_{\alpha }(\infty )\prod _{i=1}^{{n}_{\alpha }}\frac{{\omega }_{i\alpha L}^{2}-{\omega }^{2}}{{\omega }_{i\alpha T}^{2}-{\omega }^{2}},$$

where α = X, Y, Z denotes the direction of the dielectric axis; ε_α_(ω) is the dielectric function in the α direction; ω_iαT_, ω_iαL_ are the *i*th transverse optical mode and longitudinal optical mode along the α direction, respectively. Figure 3(a) plots the refractive indices of LBO measured in our experiment at 297 K and calculated at 300 K by Kurosawa relationship which is in accordance with our results. The high frequency dielectric constants ε_X_(∞) and ε_Z_(∞) of the LBO crystal at 300 K are 2.798 and 3.653, respectively. In addition, the interaction of a radiation field with the fundamental lattice vibration plays a dominating role and results in absorption of electromagnetic waves due to the creation or annihilation of lattice vibration in the far infrared region, so we also use the classical pseudo-harmonic phonon model with the first approximation^32^

2 $${\varepsilon }_{\alpha }(\omega )={\varepsilon }_{\alpha }(\infty )+\frac{{\varepsilon }_{\alpha }(st){\omega }_{\alpha }^{2}(TO)}{{\omega }_{\alpha }^{2}(TO)-{\omega }_{\alpha }^{2}-i{\gamma }_{\alpha }{\omega }_{\alpha }},$$

to further verify our experimental results. The subscript α represents the direction of the dielectric axis, ε_α_(∞) is high-frequency dielectric constant; ε_α_(st) is oscillator strength; γ is phonon damping constant. The oscillator strength is ε(st) = ε(0) − ε(∞), here ε(0) is the static dielectric constant which can be determined by means of the LST relation^31^. For the LBO crystal at 300 K, the oscillator strength ε_X_(st) and ε_Z_(st) calculated from Eq. (1) is 2.299 and 3.474, respectively. We choose the relative strong TO phonon resonances ω_X_(TO)/2π = 5.22 THz and ω_Z_(TO)/2π = 5.16 THz from Table 1 which largely influence the terahertz optical properties, as well as the phonon damping constant γ_X_/2π = 0.18 THz and γ_Z_/2π = 1.7 THz to fit the results. The theoretical fittings are shown in Fig. 3(b) and (c) which coincide with our experimental results. The absorption dispersion is very large as shown in Fig. 3(c), and an even little change in γ and ε(st) can make the theoretical results deviate from the experimental data. We are unable to employ the Kurosawa equation to calculate the refractive index due to a lack of longitudinal optical modes at liquid nitrogen temperature, so we utilized the classical pseudo-harmonic phonon model and transverse optical modes to fit the experimental results. At 80 K, the strong TO-phonon resonances are ω_X_(TO)/2π = ω_Z_(TO)/2π = 5.37 THz from Table 1. The other fitting parameters are ε_X_(st) = 2.12, ε_Z_(st) = 2.75, γ_X_/2π = 0.06 THz and γ_Z_/2π = 0.368 THz. From the theoretical fitting shown in Fig. 3(d) and (e), we can see that the two simulations are consistent with the experimental results to a large extent. The deviation in Fig. 3(e) may arise from the very low absorption at 80 K. Comparing the transverse modes B_1T_, B_2T_ at 300 K and 80 K in Table 1, we can see the wavenumber shifts of the phonon modes B_1T_, B_2T_ at these two different temperatures. Besides, with the decrease of the LBO crystal temperature, the spectral line-width becomes narrow, the profile becomes sharp, and the low frequency of the lattice vibration modes appears blue shifted. These changes may be related to broadening of the external vibration energy level at low temperature. The distinct behavior of terahertz absorption coefficient and refractive index of the LBO single crystal with temperature in these two orientations are associated with temperature-dependent characteristics of the lattice phonon modes B_1T_ and B_2T_ which are more likely due to strong anharmonicity^20, 21^. The large birefringence of the LBO crystal between the Z-axis and X-axis can be used to design terahertz devices, such as wave plates, also can be used to realize terahertz frequency conversion phase matching. In addition, due to lack of optical phonon vibration modes in the Raman spectrum of the LBO crystal when frequency is less than 3.3 THz, optical information of the LBO crystal in the terahertz range obtained by THz-TDS is very important for terahertz applications.

**Table 1:** Low-frequency Raman modes of LiB_3_O_5_ in units of THz at 300 K^20^ and 80 K^22^.

300 K	B_1*T*_ 3.3 3.96 4.8 5.22 6.66 9.84 10.86 11.4 13.14 13.8 15.12 16.02 16.5 18.24 20.22
	B_1*L*_ 3.3 4.14 4.86 5.28 6.78 9.96 10.92 11.4 13.2 13.92 15.6 16.02 16.5 19.32 20.28
	B_2*T*_ 3.3 4.56 5.16 5.4 6.78 7.38 9.3 10.08 11.58 14.1 16.44 17.22 18.18 20.22 21.18
	B_2*L*_ 3.36 4.56 5.22 5.4 6.84 7.38 9.48 10.14 11.64 14.1 16.5 17.4 18.18 20.22 21.24
80 K	B_1*T*_ 3.39 4.2 5.01 5.37 5.82 6.96 7.2 7.98 9.99 10.92 11.73 12.87 13.32 13.98 14.61 15.21
	B_2*T*_ 3.39 4.65 5.37 5.52 7.23 7.86 9.57 10.14 11.76 14.25 16.62 17.25 18.24 20.34 21.24

In conclusion, we investigated terahertz optical properties of LBO in the region of 0.2–2 THz using broadband THz-TDS. In order to study the temperature effect on the LBO crystal, the absorption coefficients and refractive indices were measured with the incident terahertz pulse polarization along the X-axis and Z-axis directions at six different temperatures among 77–297 K. The experimental results indicated that the absorption coefficient and refractive index of the LBO crystal in the X-axis and Z-axis behave differently with temperature. To explain the phenomena, we examined the behavior of the optical phonon modes at different temperatures and used the combined Kurosawa equation and classical pseudo-harmonic phonon model to demonstrate our experimental results. As a potential application, the LBO crystal can be exemplarily used as wave shapers, beam splitters, terahertz wave plates, circular polarizers and other polarization devices.

## Methods

The LBO crystal experimentally studied is commercially available at FIRSM, CAS (Fujian Institute of Research on the Structure of Matter, Chinese Academy of Sciences). It has high structural quality, excellent optical homogeneity (${\rm{\delta }}{\rm{n}}\approx {{\rm{10}}}^{-6}/\mathrm{cm}$) and high flatness (at 633 nm). The LBO crystal is 8 × 8 × 1.07 mm^3^ in size, cut along three crystallographic a-, b- and c-axis and optically finished on two of the largest faces including dielectric axis X and Z which is perpendicular to an unpolished face marked with an arrow by the supplier. The LBO crystal was experimentally characterized by broadband THz-TDS transmission measurements with a cryogenic temperature control system. In the whole experiment, the propagation of the input terahertz pulse is along the dielectric axis Y perpendicular to the two polished faces. With the help of the temperature control system, we conducted experiments at five different temperatures in both cases of terahertz polarization along X-axis and Z-axis, respectively. The measurements were performed with the LBO sample attached to a well-defined optical aperture and accommodated in a cooling experimental setup described earlier^33^. The LBO sample was screwed evenly on a metallic sample holder and the assembly was placed in a vacuum chamber with high optical transparency positioned at the center of the THz-TDS system. The vacuum chamber had a liquid nitrogen container in tight contact with the LBO sample so that effective cooling of the LBO was possible. The terahertz beam was collimated by an 8-F confocal geometry. The THz-TDS setup with a cooling system can be seen in Supplementary Fig. S1.

The amplitude transmission and the corresponding phase change are determined by $|\tilde{t}(\omega )|=|{E}_{s}(\omega )/{E}_{r}(\omega )|$ and $\phi (\omega )={\rm{\arg }}[\tilde{t}(\omega )]$, respectively, with ${E}_{s}(\omega )$ and ${E}_{r}(\omega )$ being the Fourier-transformed amplitude spectra of the terahertz pulses transmitted through the sample and reference, respectively. When an electromagnetic wave propagates through a parallel slab, the amplitude transmission $\tilde{t}(\omega )$ can be described as^32^

3 $$\tilde{t}(\omega )={t}_{12}{t}_{21}\,\exp [iL(k-{k}_{0})]\,\exp (-\alpha L/2),$$

where *t*_12_, *t*_21_ are the frequency-dependent complex Fresnel transmission coefficients; *α* is the power absorption coefficient; *k* is the sample wave vector, *k* = 2*πn*_*r*_/*λ*_0_; *k*_0_ is the vacuum wave vector, *k* = 2*πn*_*r*_/*λ*_0_; L is the sample thickness^34, 35^. The relationship of the frequency-dependent dielectric constant and refractive index of sample is as follows:

4 $$\varepsilon (\omega )={\varepsilon }_{r}-i{\varepsilon }_{i}={({n}_{r}-i{n}_{i})}^{2},$$

5 $${\varepsilon }_{r}={n}_{r}^{2}-{(\frac{\alpha \lambda }{4\pi })}^{2},\,{\varepsilon }_{i}=\frac{\alpha {n}_{r}\lambda }{2\pi },\,{n}_{i}=\frac{\alpha \lambda }{4\pi },$$

Through these relations, the power absorption coefficient and index of refraction are retrieved, respectively. The detailed setup and data extraction procedures please see Supplementary Methods.

## Electronic supplementary material

Supplementary Information
