# Supplementary material for: Temperature-dependent birefringence of lithium triborate, LBO in the THz regime
Source: Sci Rep. 2017 Aug 14;7:8122. doi: 10.1038/s41598-017-08626-2 (PMC5556023; doi:10.1038/s41598-017-08626-2)
Supplement: Supplementary file 1 — Supplementary Information [file 41598_2017_8626_MOESM1_ESM.doc]

**Supplementary Material**

**Temperature-dependent birefringence of lithium triborate, LBO in the THz regime**

**Kechao Song**^1^**, Zhen Tian**^2^**, Weili Zhang**^2,3^**, and Mingwei Wang**^1,*^

^1^Institute of Modern Optics, Nankai University, Key Laboratory of Opto-electronic Information Science and Technology, Ministry of Education, Tianjin 300350, China

^2^Center for Terahertz Waves and College of Precision Instrument and Optoelectronics Engineering, Tianjin University, and Key Laboratory of Optoelectronics Information and Technology, Ministry of Education of China, Tianjin 300072, China

^3^School of Electrical and Computer Engineering, Oklahoma State University, Stillwater, OK 74078, USA

[*wangmingwei@nankai.edu.cn](mailto:*wangmingwei@nankai.edu.cn)

**Supplementary Methods**

The measurement setup is a standard terahertz time-domain spectroscopy system based on photoconductive switch. The system is aligned into an 8-F confocal geometry by using four paraboloidal mirrors, which enables the excellent terahertz beam coupling between the transmitter and receiver and compresses terahertz beam to 2 mm at focus. The transmitter is a GaAs photoconductive switch gated by the femtosecond laser beam with a repetition rate of 88 MHz and pulse duration of 25 fs generated by Ti: sapphire self-mode-locked laser, and the detector is a ZnTe crystal module. The THz-TDS system has an effective bandwidth of 0.1–3 THz (3 mm–100 μm) and an amplitude signal to noise ratio (*S/N*) of about 2000: 1.

The 77 K cryogenic temperatures system in the experiment including a vacuum chamber, metallic sample holder, temperature display and control was designed by us. The front and rear parts of the vacuum chamber are two silicon windows that allow THz to pass through. The metallic sample holder has two identical holes to facilitate attachment of the sample and transmission of the reference pulse designated as input pulse. The temperature display and control are realized by the 325 Temperature Controller purchased from Lakeshore. Supplementary Figure S1 shows the cooling experimental setup.

The LBO sample to be characterized was placed midway between the transmitter and receiver modules at the waist of terahertz beam and screwed evenly on a metallic sample holder. The assembly was placed in a vacuum chamber with high optical transparency well positioned at the center of the THz-TDS system. The experiments were carried out at 297 K，235 K，195 K，127 K and 77 K, respectively. The direction of propagation of the input terahertz pulse was along the crystal dielectric axis Y perpendicular to the front and rear polished faces. First, the polarization was along the X-axis parallel to the unpolished surface marked with an arrow by the supplier, and the reference and sample signal was measured alternately three times at each temperature to reduce errors, then we changed the polarization to be along the Z-axis and repeated the steps above. In the transmission measurement, the transmitted electric field of the sample pulse $E_{s}(t)$ and reference pulse $E_{r}(t)$ were recorded in the time domain, and then we converted them to complex amplitude spectrum $E_{s}(\omega)$ and $E_{r}(\omega)$ respectively in the frequency domain by FFT (Fast Fourier Transform). The amplitude transmission and the corresponding phase change are determined by $\left|\tilde{t}(\omega)\right|=\left|E_{s}(\omega)/E_{r}(\omega)\right|$ and , respectively, with $E_{s}(\omega)$ and $E_{r}(\omega)$ being the Fourier-transformed amplitude spectra of the terahertz pulses transmitted through the sample and reference, respectively. Due to the limited thickness of the sample, multiple superposed pulses would be recorded in the time domain. According to the well-known amplitude transmission function of a parallel dielectric slab^1^, the complex amplitude transmission can be described as^2^

$\tilde{t}(\omega)=\frac{t_{12}t_{21}\exp\left[iL\left(k-k_{0}\right)\right]\exp(-\alpha L/2)}{1+r_{12}r_{21}\exp(-\alpha L)\exp(i2kL)}$ (1)

where $t_{12}$, $t_{21}$ are the frequency-dependent complex Fresnel transmission coefficients; $\alpha$ is the power absorption coefficient; $k$ is the sample wave vector, $k=2\pi n_{r}/\lambda_{0}$; $k_{0}$ is the vacuum wave vector, $k=2\pi/\lambda_{0}$; L is the sample thickness. Because of the relatively clean separation in time between the main transmitted pulse and the first internal reflection, the data analysis was performed on the main pulse only. For this simple case, the complex amplitude transmission is simplified as

$\tilde{t}(\omega)=t_{12}t_{21}\exp\left[iL\left(k-k_{0}\right)\right]\exp(-\alpha L/2)$ (2)

For TM polarization and normal incidence, the frequency-dependent complex Fresnel transmission coefficients are

$t_{12}=2/(\tilde{n}+1)$, $t_{21}=2\tilde{n}/(\tilde{n}+1)$ (3)

The complex refractive index of sample is

$\tilde{n}=n_{r}-in_{i}=n_{r}-i\lambda_{0}\alpha/4\pi$ (4)

The complex amplitude transmission expressed in magnitude and argument is

(5)

where $A$is the amplitude ratio, is the phase difference.

Inserting the formulas (3) and (4) into (2), simplifying it in the form of formula (5), and considering weak absorption $n_{i}/n_{r}<<1$ yields^3^

(6)

$\alpha=\frac{2}{d}\ln[\frac{4n}{A(1+n)^{2}}]$ (7)

Therefore, the power absorption coefficient and index of refraction are retrieved, respectively according to $E_{s}(\omega)$, $E_{r}(\omega)$ and thickness of sample.

**Supplementary Figures**

**Supplementary Figure S1:**

The 8-F THz-TDS setup with a standard cryostat cooling system in Oklahoma State University (OSU), USA.

**
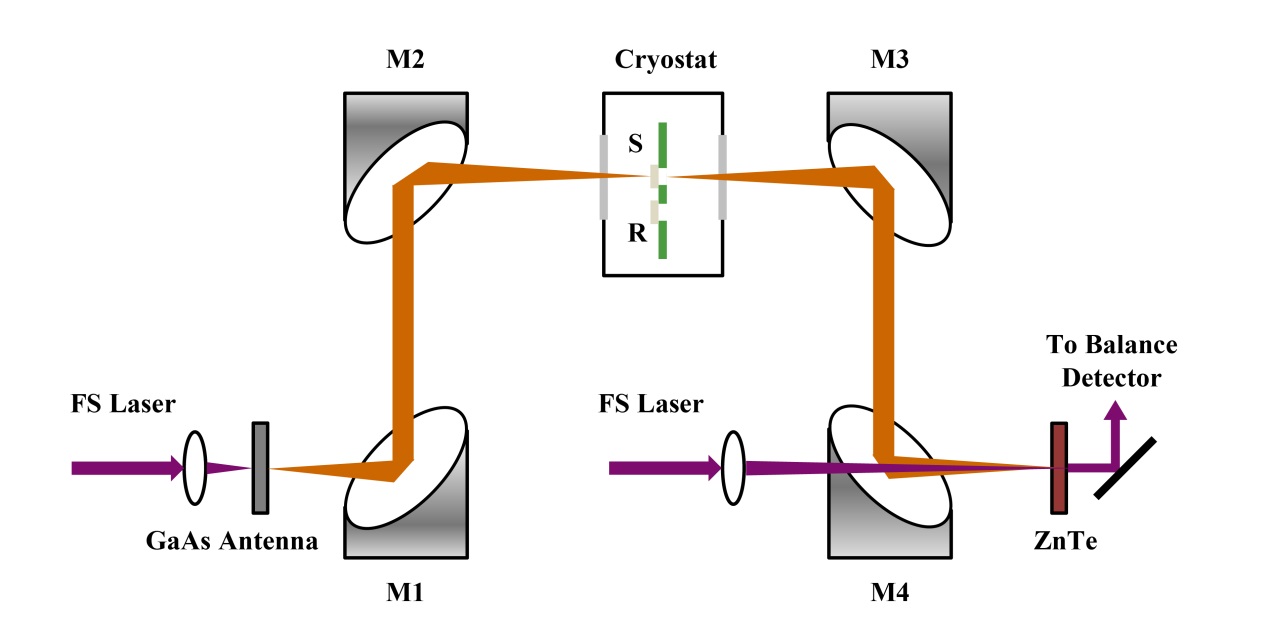
**

**Supplementary Figure S2:**

(a)-(j) Measured time and frequency domain waveforms of the reference pulse and sample pulse at 297 K, 235 K, 195 K, 127 K and 77 K, respectively. The blue solid line, the red short dashed line and the olive dashed line represent reference pulse, sample pulses polarizing along X-axis and Z-axis, respectively.


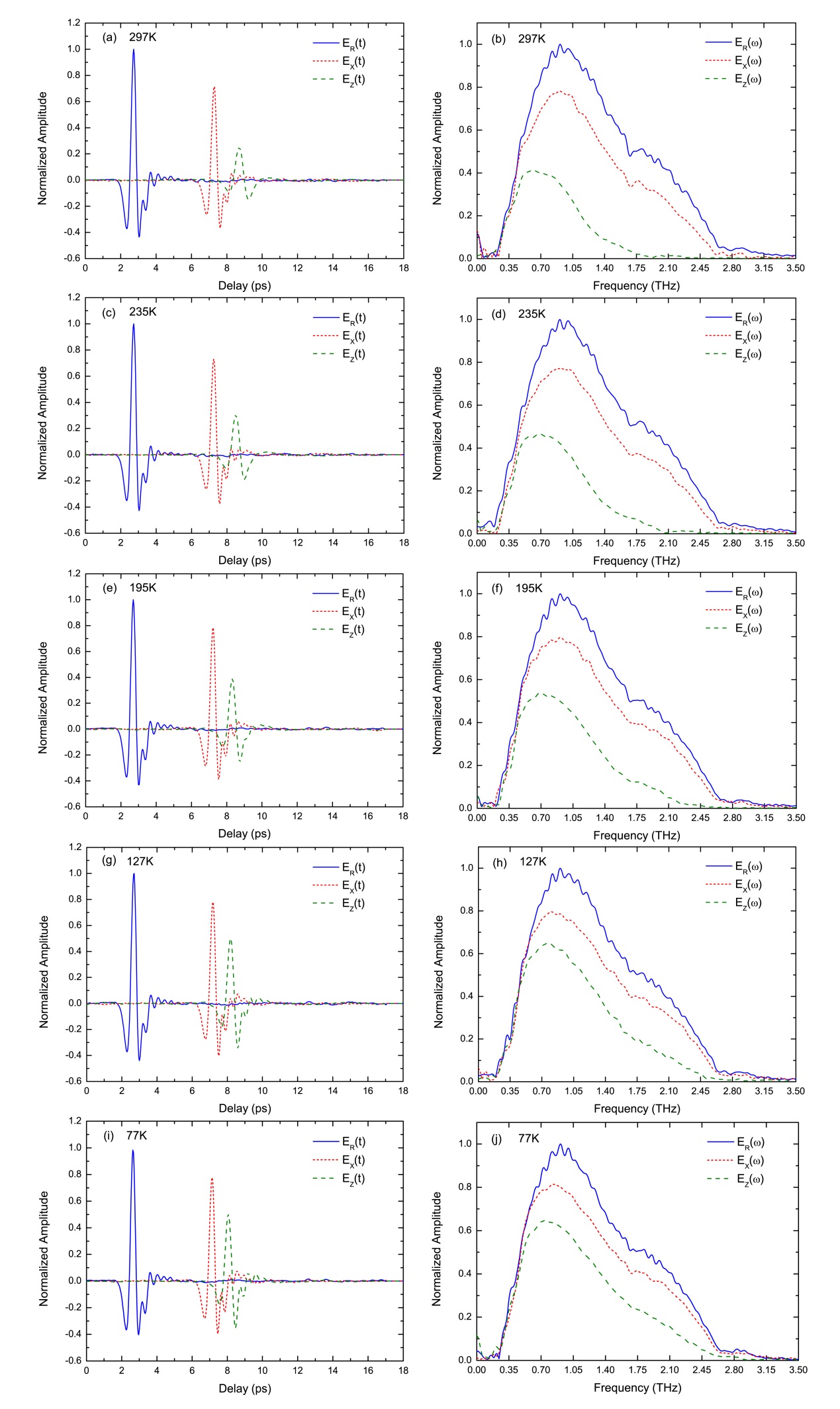


**Supplementary Tables**

**Supplementary Table S1:**

Raman modes of LiB_3_O_5_ in units of THz at 300 K^4^ and 80 K^5^.

| 300 K | $B_{1T}$ | 3.3 3.96 4.8 5.22 6.66 9.84 10.86 11.4 13.14 13.8 15.12 16.02 16.5 18.24 20.22 |
| --- | --- | --- |
|  |  | 21.84 22.92 23.4 25.62 26.88 28.62 31.86 33.72 40.08 41.7 46.74 |
|  | $B_{1L}$ | 3.3 4.14 4.86 5.28 6.78 9.96 10.92 11.4 13.2 13.92 15.6 16.02 16.5 19.32 20.28 |
|  |  | 22.08 22.92 23.4 25.68 26.88 29.82 31.86 33.72 40.08 43.26 46.74 |
|  | $B_{2T}$ | 3.3 4.56 5.16 5.4 6.78 7.38 9.3 10.08 11.58 14.1 16.44 17.22 18.18 20.22 21.18 |
|  |  | 22.08 22.92 23.46 25.68 29.16 29.58 30.66 32.46 37.8 40.14 40.92 |
|  | $B_{2L}$ | 3.36 4.56 5.22 5.4 6.84 7.38 9.48 10.14 11.64 14.1 16.5 17.4 18.18 20.22 21.24 |
|  |  | 22.14 22.92 23.58 26.46 29.16 30.12 30.78 37.2 38.7 40.26 42 |
| 80 K | $B_{1T}$ | 3.39 4.2 5.01 5.37 5.82 6.96 7.2 7.98 9.99 10.92 11.73 12.87 13.32 13.98 14.61 15.21 |
|  |  | 16.08 16.62 18.21 19.56 20.1 20.61 23.01 23.49 25.8 26.67 29.82 31.86 32.82 40.23 41.52 |
|  | $B_{2T}$ | 3.39 4.65 5.37 5.52 7.23 7.86 9.57 10.14 11.76 14.25 16.62 17.25 18.24 20.34 21.24 |
|  |  | 22.23 22.98 23.55 25.74 29.28 30.78 37.92 40.26 40.95 |

**Supplementary References**

1. Born, M. & Wolf, E. in *Principles of Optics* Ch. 1 Basic properties of the electromagnetic field, 55-65 (Cambridge University Press, 1999).

2. Zhang, W., Azad, A. K. & Grischkowsky, D. Terahertz studies of carrier dynamics and dielectric response of n-type, freestanding epitaxial GaN. *Applied Physics Letters* **82**, 2841-2843 (2003).

3. Duvillaret, L., Garet, F. & Coutaz, J.-L. Highly precise determination of optical constants and sample thickness in terahertz time-domain spectroscopy. *Applied optics* **38**, 409-415 (1999).

4. Jiang, Y. J., Wang, Y. & Zeng, L. Z. Analysis of Raman spectra of LiB3O5 single crystals. *Journal of Raman spectroscopy* **27**, 601-607 (1996).

5. Xiong, G., Lan, G., Wang, H. & Huang, C. Infrared reflectance and Raman spectra of lithium triborate single crystal. *Journal of Raman spectroscopy* **24**, 785-789 (1993).
